# Optimizing approaches for targeted integration of transgenic cassettes by integrase-mediated cassette exchange in mouse and human stem cells

**DOI:** 10.1093/stmcls/sxae092

**Published:** 2025-01-08

**Authors:** Phalguni Rath, Philipp Kramer, Daniel Biggs, Chris Preece, Nicole Hortin, Rebeca Diaz, Marta Perez-Alcantara, Xiang Li, Arnaud Bolard, Nicola Beer, Mark McCarthy, Benjamin Davies

**Affiliations:** Wellcome Centre for Human Genetics, University of Oxford, Oxford OX3 7BN, United Kingdom; Wellcome Centre for Human Genetics, University of Oxford, Oxford OX3 7BN, United Kingdom; Wellcome Centre for Human Genetics, University of Oxford, Oxford OX3 7BN, United Kingdom; Wellcome Centre for Human Genetics, University of Oxford, Oxford OX3 7BN, United Kingdom; Wellcome Centre for Human Genetics, University of Oxford, Oxford OX3 7BN, United Kingdom; Wellcome Centre for Human Genetics, University of Oxford, Oxford OX3 7BN, United Kingdom; Wellcome Centre for Human Genetics, University of Oxford, Oxford OX3 7BN, United Kingdom; Genetic Modification Service, Francis Crick Institute, London NW1 1AT, United Kingdom; Wellcome Centre for Human Genetics, University of Oxford, Oxford OX3 7BN, United Kingdom; Wellcome Centre for Human Genetics, University of Oxford, Oxford OX3 7BN, United Kingdom; Wellcome Centre for Human Genetics, University of Oxford, Oxford OX3 7BN, United Kingdom; Wellcome Centre for Human Genetics, University of Oxford, Oxford OX3 7BN, United Kingdom; Genetic Modification Service, Francis Crick Institute, London NW1 1AT, United Kingdom

**Keywords:** integrase, cassette exchange, safe-harbor locus, CRISPR-activation, CRISPR-inhibition

## Abstract

To enable robust expression of transgenes in stem cells, recombinase-mediated cassette exchange at safe harbor loci is frequently adopted. The choice of recombinase enzyme is a critical parameter to ensure maximum efficiency and accuracy of the integration event. We have explored the serine recombinase family of site-specific integrases and have directly compared the efficiency of PhiC31, W-beta, and Bxb1 integrase for targeted transgene integration at the *Gt(ROSA)26Sor* locus in mouse embryonic stem cells. All 3 integrases were found to be suitable for efficient engineering and long-term expression of each integrase was compatible with pluripotency, as evidenced by germline transmission. Bxb1 integrase was found to be 2-3 times more efficient than PhiC31 and W-beta. The Bxb1 system was adapted for cassette exchange at the *AAVS1* locus in human induced pluripotent stem (iPS) cells, and the 2 commonly used ubiquitous promoters, CAG and Ef1α (*EIF1A*), were tested for their suitability in driving expression of the integrated transgenic cargo. *AAVS1-*integrated Ef1α promoter led to a very mosaic pattern of expression in targeted hiPS cells, whereas the *AAVS1-*integrated CAG promoter drove consistent and stable expression. To validate the system for the integration of functional machinery, the Bxb1 integrase system was used to integrate CAG-driven CRISPR-activation and CRISPR-inhibition machinery in human iPS cells and robust sgRNA-induced up- and downregulation of target genes was demonstrated.

## Graphical Abstract

**Graphical Abstract:**
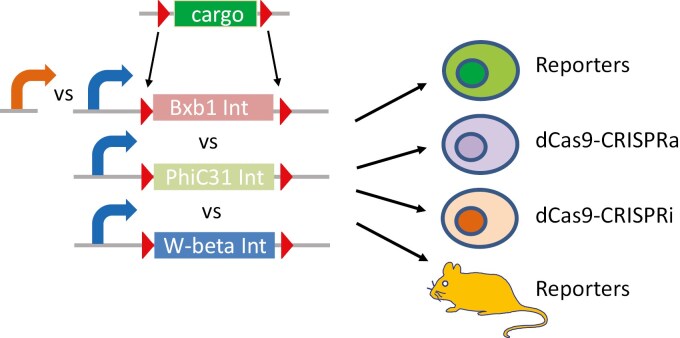

## Introduction

A number of approaches have been employed to equip stem cell lines with machinery, enabling expression of reporter genes or CRISPR machinery for subsequent functional studies. The simplest approaches rely on random integration of a promoter-driven construct using either stable transfection^1^ or lentiviral transduction.^2^ Random integration methods, however, can lead to mutagenesis events at the genomic insertion sites^3^ and/or position effects can result in dysregulation and silencing of the transgenic cargo.^4^ To address these concerns, so called safe harbor loci have been employed in both mouse^5^ and human stem cells,^6^ at which integration of a transgenic construct is not associated with any detrimental effects and where the expression characteristics of the transgene are preserved. In mouse embryonic stem (ES) cells, the *Gt(ROSA)26Sor* locus is frequently employed^7^ and in human-induced pluripotent stem (iPS) cells, the *AAVS1* locus is a common safe harbor integration site.^8^

Targeted insertion into these safe harbor loci has been achieved by conventional^7^ or CRISPR/Cas9-assisted gene targeting.^9,10^ Despite the relatively high frequency of targeted integration, the associated validation of the integration events can be time-consuming, particularly when using CRISPR/Cas9-assisted manipulations,^11^ where concatemerization of the targeting vector,^12^ large deletions,^13^ and rearrangements at the target site^14^ must be ruled out.

An alternative strategy is the use of site-specific recombinases to enable an enzyme-driven integration of transgenic cargo into safe harbor loci. These protocols involve 2 steps: firstly, the integration of a docking site at the safe harbor locus, comprising the site-specific recombinase target site (lox and FRT sites in the case of the Cre and Flp tyrosine recombinases,^15,16^ respectively, or site-specific attachment (att) sites for the integrase family of serine recombinases^17^); followed by a second step involving the recombinase-driven integration of the transgenic cargo, delivered as an exchange vector harboring the cognate site-specific recombinase sites. Frequently, 2 sets of recombinase target sites are employed flanking the docking site/transgenic cargo, allowing a direct exchange of sequence in a manipulation known as recombinase-mediated cassette exchange (RMCE).^18^ For particularly commonly used stem cells lines, the RMCE approach is useful as lines harboring docking sites can be prepared and then used for integration of numerous different transgenic cargos, facilitating production and allowing comparative studies of variant transgenes.

For an optimal RMCE protocol, efficiency, accuracy, and a resulting robust and consistent transgene expression are important parameters. In this study, we perform a side-by-side comparison of the efficiency of different integrases for RMCE at the *Gt(ROSA)26Sor* locus in mouse ES cells, comparing the commonly used PhiC31^17^ and 2 further serine integrases which have been shown to be active in mammalian cells, the Bxb1 and W-beta integrases from the eponymous bacteriophages of *Mycobacterium smegmatis* and *Bacillus anthracis*, respectively.^19,20^ We find prolonged expression of both integrases in mouse stem cells to be compatible with retained pluripotency, as evidenced by germline transmission data and find Bxb1 integrase to be 2-3 fold as efficient as PhiC31 and W-beta integrases.

We further explore the utility of the Bxb1 RMCE system for achieving expression of transgenes at the *AAVS1* safe harbor locus in human iPS cells. Although the use and functionality of the CAG promoter are well proven at the *Gt(ROSA)26Sor* locus in mice,^21^ the literature surrounding the most appropriate ubiquitous promoter in human stem cells identifies both the CAG^10,22^ and the EF1α^23-25^ promoter. Here, we compare transgene expression following Bxb1 integrase RMCE at the *AAVS1* locus and find the CAG promoter to provide more consistent and reliable expression. Proof-of-concept human iPS cell lines harboring CRISPR-activation and CRISPR-inhibition machinery generated using the Bxb1 RMCE system are presented.

## Results

### Comparison of integrase efficiency for RMCE

**Figure 1.**
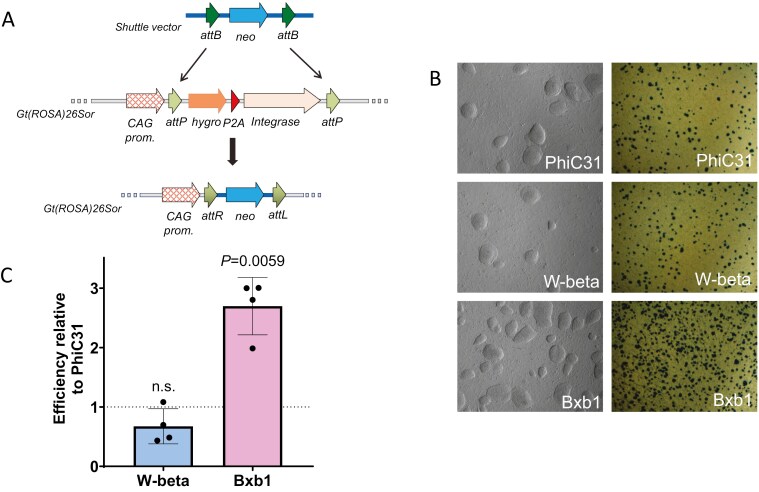
Comparative efficiencies of PhiC31, Bxb1, and W-beta integrases. (A) Strategy used for the comparison of integrase efficiency using a CAG driven Hygro-P2A-Integrase cassette which is exchanged for neomycin resistance by the action of the integrase. (B) Photomicrographs of neomycin resistant colonies (Left hand panel is phase contrast, Right hand panel is low magnification view of colonies stained with methylene blue). (C) Relative efficiency of W-beta and Bxb1 integrase, normalized to PhiC31 integrase. Histograms show the mean relative efficiency across 4 independent transfections and the error bars represent 1 SD. Statistical comparisons against the theoretical mean of 1 were performed with a 1 sample *t*-test.

Docking sites for integrase-mediated cassette exchange using 3 different serine recombinases (integrases), PhiC31, Bxb1, and W-beta, were targeted to the *Gt(ROSA)26Sor* locus in JM8F6 ES cells (C57BL/6N) using conventional gene targeting. Each docking site comprised a CAG promoter driving the expression of both a hygromycin resistance cassette and a codon-optimized integrase, separated by a P2A sequence to allow bicistronic expression. Flanking the hygromycin resistance-P2A-integrase open reading frame, the relevant integrase attP sites were positioned (Supplementary Figure S1). The docking site cell lines B03 (PhiC31), A06 (W-beta), and C11 (Bxb1) were selected for functional work and were confirmed as being heterozygous with a single copy confirmed by qPCR (Supplementary Figure S1C and D). These cell lines were transfected with simple exchange vectors, which comprised a promoterless neomycin resistance cassette flanked by the respective integrase attB sites (Figure 1A).

Expression of the integrase from the docking site led to recombination between the genomic attP sites and the attB sites on the exchange vector, resulting in an exchange of the intervening sequence, incorporating the promoterless neomycin resistance cassette downstream of the CAG promoter,^26^ and thus rendering the cell lines resistant to G418 (Figure 1B; Supplementary Figure S2). The integrase-mediated cassette exchange thus led to the targeted insertion of the neomycin resistance cargo at the *Gt(ROSA)26Sor* locus with the concomitant removal of the integrase expression cassette within the docking site.

Since no neomycin resistant clones were recovered which were not positive for the 5’ integration PCR (Supplementary Figure S2), an assessment of the number of G418 resistant colonies provided a proxy for the relative activity of the 3 integrases (Figure 1C). Bxb1 outperformed PhiC31 and W-beta by 2-3-fold. Sequencing of candidate clones revealed the 5’ and 3’ recombination to have occurred accurately, with the 5’ attP x attB recombination event leading to an attL site and the 3’ attP × attB recombination event leading to an attR site for the respective integrase. There was no evidence for any site damage occurring in any of the 16 clones analyzed (Supplementary Figure S3). Analysis of clones to assess the copy number of the neomycin resistance cassette following successful RMCE revealed a stable single copy number in all clones analyzed (Supplementary Figure S4).

### Validation of pluripotency and proof-of-concept animal model generation

**Figure 2.**
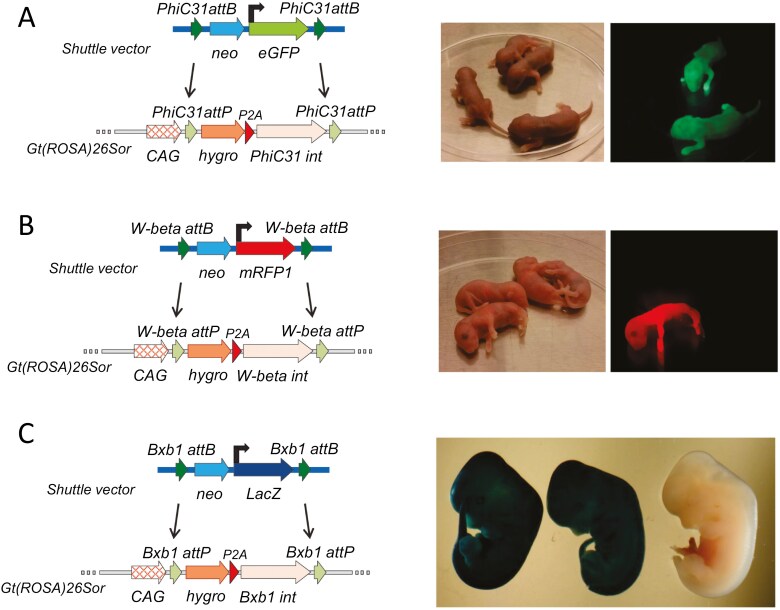
PhiC31, W-beta, and Bxb1 integrase expression is compatible with germline transmission. Proof-of-concept integration of (A) a CAG promoter driven eGFP cassette (PhiC31), (B) a CMV promoter driven mRFP1 cassette (W-beta), and (C) a CAG promoter driven LacZ cassette (Bxb1). Left-hand panels shows the recombinase-mediated cassette exchange strategies, and the right-hand panel demonstrates the germline transmission of the targeted alleles, by fluorescent imaging of F1 offspring at P1 (A and B) or lacZ staining of F1 embryos at E11.5.

In order to validate the pluripotency of the integrase RMCE ES cells, their ability to contribute to the germline of chimeras was investigated. In particular, this robust test of pluripotency was necessary to rule out the possibility that the constitutive integrase expression used in our strategy might compromise pluripotency and thus their utility for functional studies and mouse line production. Subsequently, for the 3 integrase systems, recombinant ES cells were generated in which 3 different reporter constructs (each with their own promoter) were successfully integrated by the 3 different integrase-mediated cassette exchanges. A CAG promoter-driven GFP fluorescent cassette was used for the PhiC31 system (Figure 2A), a CMV promoter-driven mRFP1 fluorescent cassette was used for the W-beta system (Figure 2B), and a CAG promoter-driven LacZ reporter cassette was used for the Bxb1 system (Figure 2C). These recombinant ES cells were injected into blastocysts and the resulting chimeras were bred to assess germline transmission. In all 3 cases, successful germline transmission was seen as evidenced by reporter expression in the subsequent F1 offspring, confirming that integrase expression did not compromise the pluripotency of the ES cell lines (Figure 2A-C).

### Adaptation of the Bxb1 system for RMCE at AAVS1 in human iPS cells

**Figure 3.**
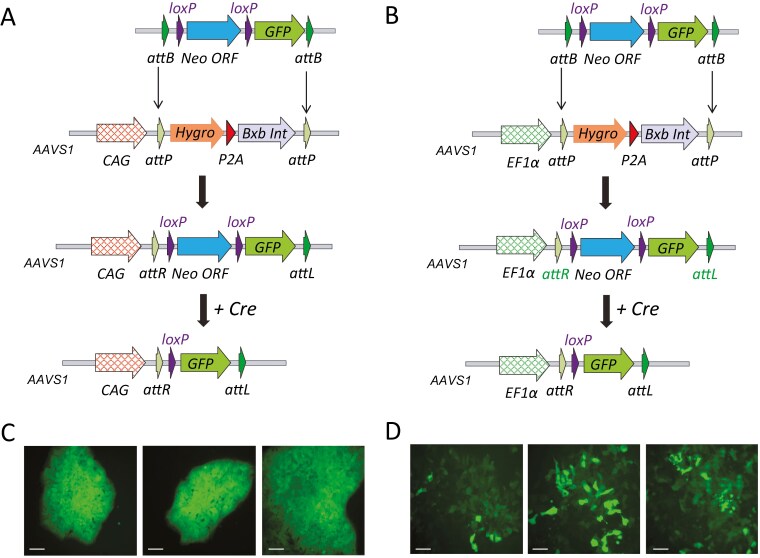
CAG and EF1α promoter-driven expression at the *AAVS1* locus in iPS cells. Recombinase-mediated cassette exchange strategy for the integration of a promoterless GFP with a loxP flanked promoterless Neomycin resistant cassette into 2 different iPS cells lines with landing pads for Bxb1 integrase exchange targeted to the AAVS1 locus, driven by either the CAG promoter (A) or the EF1α promoter (B). Bottom panel shows the consequence of Cre deletion of the Neomycin cassette and the activation of the GFP expression. Example photomicrographs of GFP fluorescence in the resulting colonies are shown for the CAG promoter (C) and the EF1α promoter (D). (100-μm scale bar).

Having confirmed that the most efficient RMCE was achieved by the Bxb1 integrase and that the expression of the integrase did not compromise pluripotency, we were keen to establish an analogous system for transgene integration into the human safe harbor locus, *AAVS1*, in human iPS cells. A landing pad and exchange vector system was devised in which a strong ubiquitous promoter drives the expression of the selection cassette and the Bxb1 integrase. If an exchange vector is used with a loxP flanked Neomycin selection cassette, with a downstream open-reading frame of the transgene of interest, the system allows for Cre recombinase activatable gene expression (Figure 3A and B). Following RMCE and Cre recombinase-mediated deletion of the Neomycin cassette, the expression of the transgene of interest is then linked to the ubiquitous promoter at the landing pad.

In mouse, the CAG promoter is well established for achieving robust ubiquitous expression when used at the *Gt(ROSA)26Sor* locus;^27^ in human iPS cells, 2 ubiquitous promoters are frequently used to drive expression, the CAG promoter^26^ but also the EF1α promoter from the human eukaryotic translation elongation factor 1α1 (*EEF1A1*). Subsequently, 2 identical systems for Bxb1 integrase-mediated cassette exchange at *AAVS1* were built, but with these 2 different promoter driving the selection/integrase machinery to allow a direct comparison of the promoters’ behavior when integrated at single copy into *AAVS1* (Figure 3; Supplementary Figures S5 and S6).

An exchange vector was designed which incorporated a loxP flanked promoterless neomycin selection cassette, followed by a promoterless GFP, all flanked by Bxb1 attB sites. Following transfection of the human iPS cells harboring either of the docking sites (C13 [CAG promoter], C10 [EF1α promoter]), G418 resistant colonies were obtained which were correctly integrated at both the 5’ and 3’ ends (Supplementary Figure S7). A more visible assessment of the stability of the promoter activity was obtained by transient Cre transfection of iPS cell clones into which the GFP reporter had correctly integrated (clone #4 [CAG promoter] and clone #10 [EF1α promoter]). The expression of the promoterless GFP is activated by Cre-recombinase-mediated deletion of the Neomycin cassette, leading to active GFP transcription from the integrated CAG or EF1α promoter (Figure 3A and B). Interestingly, GFP expression driven from the EF1α promoter at *AAVS1* appeared highly variable, whereas the GFP expression driven from the CAG promoter at *AAVS1* was robust and uniform (Figure 3C and D). To rule out any artifactual assessment due to mosaicism of the Cre recombinase reaction, individual clones were expanded, the recombination event confirmed by PCR (clones C3 and C5 [EF1α promoter] and clones C4 and C72 [CAG promoter]; Supplementary Figure S7) and these individual clones were then plated at low density to assess GFP expression. Again, highly variable GFP expression was evident when the EF1α promoter was used, whereas stable expression of GFP was observed when the CAG promoter was used (Supplementary Figure S8).

To test the reproducibility of the approach, the CAG-driven Bxb1 RMCE system was introduced into the *AAVS1* locus of a further human iPS cell line (SBAd3-4) and stable integration of a conditionally activatable GFP and mRFP1 fluorescent reporter was achieved in this independent cell line. Incubation of the cultures with a cell permeable Cre recombinase activated the expression of the 2 fluorophores (GFP and mRFP1) from the integrated CAG promoter and low-density plating of these clones resulting in stable and robust expression (Supplementary Figure S9). Pluripotency analysis of the docking site iPS cell line was confirmed as being similar to that of the parental SBAd3-4 line (Supplementary Figure S10).

### Using the Bxb1 RMCE system for equipping iPS cell lines with CRISPR/Cas9 machinery

The previous GFP experiment demonstrated that the CAG promoter leads to stable and uniform expression in undifferentiated iPS cells and that a floxed neomycin selection cassette is sufficient to prevent read-through translation of the downstream GFP cargo. The system thus lends itself to Cre-activable expression of any type of transgene.

As proof-of-concept, we tested the system for equipping human iPS cells with CRISPR/Cas9 machinery for CRISPR-activation (CRISPRa) and CRISPR-inhibition (CRISPRi) of target genes. Preparing cell lines with this CRISPR/Cas9 machinery would potentially enable the modulation of gene expression from their native promoters by transfection of the resulting cell lines with sgRNAs.

**Figure 4.**
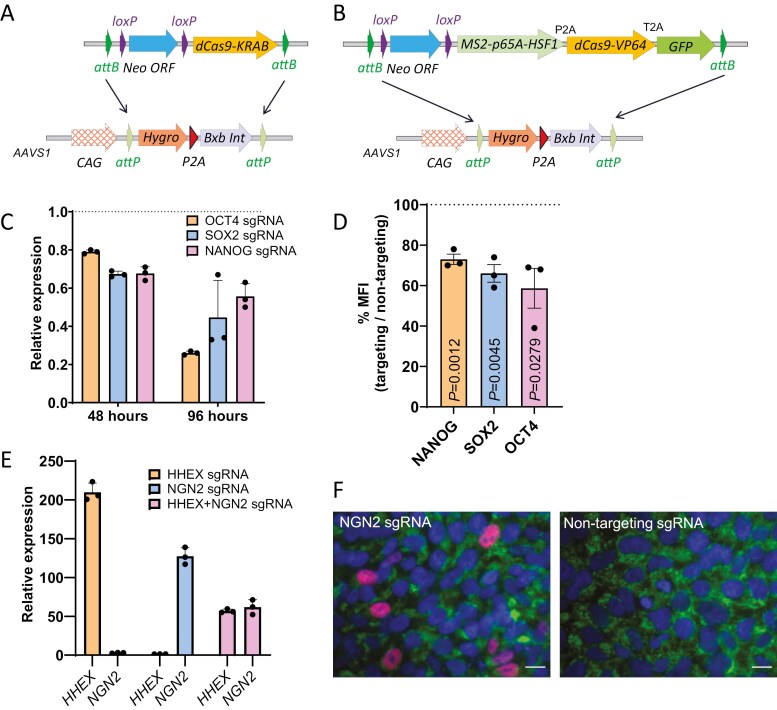
Proof of concept *AAVS1* targeted cell lines expressing CRISPRi and CRISPRa machinery. Strategy for targeted integration of a Cre-activatable dCas9-KRAB (CRISPRi) (A) and dCas9-SAM (CRISPRa) (B) at the *AAVS1* locus. Relative mRNA expression levels of *OCT4*, *SOX2*, and *NANOG* 48- and 96-hours posttransfection (C) and relative proportion of median fluorescence intensity of FACS quantification for OCT4, SOX2, and NANOG protein expression (D) of the KOLF-AAVS1-KRAB iPS cells with sgRNAs designed against the *OCT4*, *SOX2*, and *NANOG* promoter, relative to a nontargeting sgRNA. Statistical comparisons against the theoretical mean of 1 were performed with a 1 sample *t*-test. (E) Expression levels of *HHEX* and *NGN2* 48-hours posttransfection of the KOLF-AAVS-SAM iPS cells with sgRNA against *NGN2* and/or *HHEX*, relative to a nontargeting sgRNA. (F) Immunohistochemical staining of *NGN2* (red), TRA-1-81 (green), and DAPI (Blue) for KOLF-AAVS-SAM cells transfected with either the *NGN2* sgRNA or a nontargeting sgRNA (50-μm scale bar).

For CRISPRi, an exchange vector was prepared with the loxP flanked promoterless neomycin resistance cells upstream of the open-reading frame of a dCas9-KRAB fusion protein,^28^ flanked by Bxb1 attB sites (Figure 4A). For CRISPRa, a similar structure of exchange vector was used, but driving the expression of the CRISPR/Cas9 effector used for the Synergistic Activation Mediator (SAM) system^29^ (Figure 4B). Specifically, the MS2-p65-HSF1 component was expressed together with the dCas9-VP64 component separated by a P2A peptide to allow bicistronic expression.

Following successful Bxb1 integrase RMCE, recombinant clones were treated with Cre recombinase and individual subclones were isolated and screened for the excision of the floxed neomycin resistance cassette and the activation of the CRISPRa / CRISPRi machinery, creating cell lines which were named KOLF-AAVS-SAM (CRISPRa) and KOLF-AAVS-KRAB (CRISPRi). Normal expression of pluripotency markers and a euploid karyotype were confirmed for these 2 cell lines (Supplementary Figure S11A-D). The heterozygous status of the 2 iPS cell lines was confirmed by PCR of the wild-type *AAVS1* locus and by qPCR analysis (Supplementary Figure S11E and F). In addition, the stability of dCas9 expression was confirmed following extended passaging (Supplementary Figure S11G and H).

### CRISPRa and CRISPRi functionality

To test the functionality of the CRISPRi system in the engineered KOLF-AAVS-KRAB cells, sgRNAs addressing the proximal promoters of the pluripotency factors *NANOG*, *POU5F1* (*OCT4)*, and *SOX2* along with a control nontargeting sgRNA were synthesized and delivered by transient transfection. Downregulation of the target genes at the RNA level was confirmed by quantitative PCR (Figure 4C) and, at the protein level by flow cytometry (Figure 4D), comparing expression following transfection of a targeting to a nontargeting sgRNA.

For the functionality of the CRISPRa system in the engineered KOLF-AAVS-SAM cells, sgRNAs with 2 MS2 aptamer sequences were designed against the proximal promoters of *NGN2* and *HHEX*, along with a nontargeting sgRNA, synthesized as RNAs by in vitro transcription and were transfected into the KOLF-AAVS1-SAM cell line. Upregulation of the target genes’ expression was confirmed 24-72 hours after transfection by quantitative PCR (Figure 4E) and, for *NGN2* where an antibody was available, at the protein level by immunohistochemistry (Figure 4F).

The functionality of these CRISPR-effector systems confirmed the validity of the Bxb1 RMCE methodology for equipping cell lines with CRISPR/Cas9 machinery.

## Discussion

We have demonstrated the utility of the Bxb1 integrase for RMCE at safe harbor loci and have optimized the design of RMCE landing pads to enable transgene expression to be directed ubiquitously in stem cells. The landing pad design includes the constitutive expression of the integrase, so that, upon integration of the transgenic cargo, it catalyzes its own excision, avoiding the need for ectopic supply of the integrase. This streamlined integration of transgenic cargo into safe-harbor loci presents an alternative to CRISPR-assisted gene targeting and thus avoids the challenges associated with screening for CRISPR induced aberrations, such as off-target mutagenesis,^30^ on-target structural rearrangements,^11-14^ and loss of p53.^31^

Bxb1 is well established as an efficient integrase enzyme suitable for targeted integration into the mammalian genome.^19,32,33^ The efficiency has been found to be sufficiently high to permit the integration of transgenes into mouse oocytes harboring a suitable landing pad without any kind of selection.^34^ The high efficiency of the enzyme has also allowed its application at scale in HEK293T cells for parallel functional genetic assays, where a library of sequences can be integrated efficiently in a cell population.^35-37^

Comparative studies have also concluded that Bxb1 integrase outcompetes other members of the serine integrase family in terms of efficiency, in agreement with our results.^19,20^ One recent study compared PhiC31 and Bxb1 RMCE strategies for large payload integration at the *AAVS1* locus in hiPS cells and found the Bxb1 integrase to be ~10× more efficient.^38^

Concern has been raised about the activity of the integrases at sites within the genome with a degree of homology to the attP or attB sites. Indeed, these so-called pseudo sites have been used intentionally for the integration of therapeutic transgenes.^39,40^ We did not explore our cell lines for any off-target integration, but the selection strategy used requires integration into an expressed sequence which might limit these events. Furthermore, the quantitative PCR analysis of copy number of integrating constructs in the mouse ES cell study suggests single copy integration in the tested clones. One previous study, however, has reported some degree of off-target integration for the W-beta integrase in CHO cells,^20^ perhaps making this integrase unsuitable for precise genome engineering.

Previous studies employing Bxb1 integrase for RMCE for transgenic cargo insertion at safe harbor loci in mouse oocytes^34^ or in human iPS cells^41^ have suggested the enzyme to be safe. If the enzyme were active at pseudo sites, one might expect karyotypic anomalies and loss of developmental capacity. The constitutive expression of Bxb1 integrase that is inherent in our landing pad design provides further evidence for its safety, as this prolonged expression of the integrase did not appear to compromise the iPS cell pluripotency or induce any gross genome instability, and the constitutive expression of all 3 tested integrases in mouse ES cells was compatible with germline transmission in vivo.

For constitutive expression in the mouse, the CAG promoter was used for the docking site, consistent with previous reports concerning the reliability of this promoter when positioned at the *Gt(ROSA26)Sor* locus in mouse.^21,27^ In human iPS cells, the evidence of the behavior of CAG when integrated at the *AAVS1* locus is somewhat confused, with some studies concluding consistent expression in pluripotent iPS cells^10,42,43^ but others reporting variable expression due to DNA methylation even in the pluripotent state.^44^ Similarly, the EF1α promoter was identified as the most stable promoter for driving transgene expression in human ES cells when randomly integrated,^45^ yet another study concluded variable expression when integrated at the *AAVS1* locus in iPS cells.^22^

These conflicting results may result from differences in the CAG and EF1α promoter, with both longer and shorter versions of the promoter being adopted. A short ~230 bp EF1α promoter comprising the proximal promoter and the 5' untranslated region of the *EEF1A1* gene has been used,^22^ but also a longer ~1.2 kb promoter including the first intron and the downstream intron/exon boundary is reported.^46^ Similarly, the original ~1.7 kb CAG promoter, comprising the cytomegalovirus immediate early enhancer, the chicken beta-actin promoter, exon 1, and intron 1 followed by a rabbit beta globin intron/exon boundary,^26^ is sometimes shortened with the truncation of the intron^47^ or the promoter is sometimes confused with the related promoters, CBA and CBh, which have also replaced portions of the chicken beta-actin intron with viral intronic sequences.^48,49^ Whether these modifications alter the stability of the promoters when integrated at single copy into the mammalian genome remains to be formally tested. An additional factor which might influence expression from the integrated promoter could be the presence of the extra attR sites that are positioned between the promoter and the downstream transgenic cargo, following the RMCE event. Whether these small viral sequences influence promoter stability, with the Ef1α promoter being proportionally more affected, remains unclear.

The results of our side-by-side analysis of identical RMCE systems at *AAVS1* would strongly favor the 1.7-kb CAG promoter for ubiquitous, stable expression in iPS cells in their pluripotent state. Clear evidence for silencing was seen when using the larger 1.2-kb EF1α promoter. What remains to be tested is the stability of the CAG promoter-driven expression following differentiation, as there are many reports of silencing when iPS cells are differentiated.^44^ However, there are encouraging reports of maintained expression of *AAVS1* integrated CAG promoter-driven constructs in cardiomyocytes^22,50^ and during in vivo differentiation to teratoma.^50^

The proof-of-concept iPS cell lines generated with the Bxb1 integrase RMCE system showcase the simplicity of integrating transgenic cargo for ubiquitous expression. In their own right, the CRISPRa/i lines generated showed robust gene up and down regulation using simple sgRNA transfection, creating a very simple experimental system for functional gene analysis in stem cells, complementing a number of published iPS cell lines expressing functional CRISPRa and CRISPRi machinery.^51-54^ A further application of these cell lines would be to modulate the expression of key differentiation transcription factors to stimulate differentiation down a particular lineage, as was demonstrated for CRISPRa-induced NGN2 upregulation for the stimulation of neuronal differentiation.^55^ Although the CRISPR machinery in the cells was found to be stably expressed in pluripotent stem cells following long-term passaging, CRISPR machinery has been found to be susceptible to silencing, particularly following differentiation.^56^ Whether the CRISPRa/I lines retain their functionality through differentiation remains to be explored and will be addressed in future studies. It is of note, that the CRISPRi line reported in our study has already been used successfully for CRISPR-interference in iPS cell-derived macrophages, suggesting some stability of expression.^57^

In summary, we demonstrate that Bxb1 integrase provides a versatile, simple, and safe route for targeted integration into the genome of stem cells at high efficiency. In addition, we show that ubiquitous expression of Bxb1, PhiC31, and W-beta integrases in stem cells simplifies RMCE manipulations within the genome of stem cells and is compatible with pluripotency. Lastly, we present data suggesting that the CAG promoter at *AAVS1* is more reliable for driving functional machinery than *EF1a* at *AAVS1*, in terms of consistency of expression. The disadvantage of the integrase approach is the necessity for a landing pad to be generated; however, recent development with CRISPR prime editing systems have enabled the introduction of integrase att sites into the genome,^58^ and fusion of the prime editors with Bxb1 integrases has allowed targeted integration with a single enzyme.^59^ Interestingly, recent genomic scans of clinical and environmental bacterial isolates have revealed novel integrase family members, which when tested in mammalian cells exceed the efficiency of Bxb1,^60^ pathing the way for more efficient manipulations of the genome.

## Methods

### Construction of targeting vectors for landing pads and exchange vectors for targeted integration

The *Gt(ROSA26)Sor* targeting vectors used to target the Bxb1, PhiC31, and W-beta RMCE landing pads were generated by modifying pROSA26.10 (hygro attP) (a kind gift from Ralf Kuehn^61^) which contained homology arms, a diphtheria toxin A chain (dtA) negative selection cassette and a PGK driven hygromycin resistance cassette. A CAG promoter driven hygromycin resistance cassette followed by a P2A-Integrase expression cassette was cloned between the 2 homology arms, and attP sites for the 3 tested integrase were incorporated immediately upstream and downstream of the hygro-P2A-Integrase open-reading frame.

For the CAG promoter *AAVS1* targeting vector, the CAG-Bxb1 attP-Hygro-P2A-Bxb1 integrase-Bxb1-attP array was subcloned from the above *Gt(ROSA26)Sor* vector between the homology arms in an *AAVS1* targeting vector (Addgene #22075), replacing the SA-T2A-Puromycin resistance cassette originally in this vector. For the EF1α promoter *AAVS1* targeting vector, the above vector was modified by excising the CAG promoter and substituting a PCR generated EF1α promoter fragment, obtained from dCas9-VP64-T2A-GFP plasmid (Addgene #61422).

For the exchange vectors, synthetic linkers were generated harboring 2 attB sites for the 3 tested integrases and a loxP flanked promoterless neomycin resistance cassette was cloned between these attB sites. For the reporter exchange vectors, either a CAG promoter-driven eGFP or LacZ cassette or a CMV promoter-driven mRFP1 cassette was subcloned downstream of the loxP flanked neomycin resistance cassette. For the CRISPRa exchange vector, the MS2-p65A-HSF1 cassette (Addgene #61423) was linked via a P2A cassette to dCas9-VP64-T2A-GFP (Addgene #61422) to create a single open reading frame expressing all components of the CRISPR-based SAM system^29^ and cloned between the Bxb1 attB sites. For the CRISPRi exchange vector, the dCas9-KRAB cassette^28^ (Addgene #50919) was subcloned between the Bxb1 attB sites.

### Culture and gene targeting of human iPS cells

KOLF2-C1 (WTSIi018-B-1; RRID:CVCL_9S58) human iPS cells were cultured in Essential8 media (Thermofisher Scientific, #A1517001) on a Vitronectin substrate (Gibco, #A14700). SBAd3-4 (STBCi322-A; RRID:CVCL_ZX55) human iPS cells were cultured in mTeSR1 media (Stemcell Technologies) on a Matrigel substrate. Cells were dissociated with either TrypLE Select (Thermofisher Scientific, #12563011) or ReLeSR (Stemcell Technologies, #05872) for 5 minutes and replated in media supplemented with 5 μM Y-27632 (R&D Systems, #1254/10).

In single suspension, 1 × 10^6^ cells were mixed with 550 ng of targeting vector and 450 ng of pX330-Puro-AAVS1 (a modified version of pX330 [Addgene #42230] with the addition of a *Pgk1* promoter driven Puromycin resistance cassette) and resuspended in 110 μL of solution R (Thermofisher Scientific). Cells were electroporated using the Neon transfection system (Thermofisher Scientific) (1200 V, 30 ms, 2 pulses), selected in 350-μg/mL Puromycin for 48 hours, and expanded for clonal isolation in 200-μg/mL Hygromycin. Resistant colonies were manually picked, expanded, replica plated, and screened for homologous recombination events using primers, AAVS-Bxb-F1 and CMV-IER (for the CAG allele) or EF1a-R1 (for the EF1α allele) to detect integration at the 5' end and primers, SV40-pA-F and AAVS-Bxb-R1, to detect integration at the 3' end. Targeted clones were screened for the expression of pluripotency markers using the Stemflow Human Pluripotent Stem Cell Transcription Factor Analysis Kit (BD Biosystems, #560589), analyzed with the LSRFortessa X-20 (BD Biosciences) and quantified by FlowJo software (BD Biosciences, version 10.6.2).

### Culture and gene targeting of mouse ES cells

JM8.F6 (RRID:CVCL_J961) mouse ES cells were cultured in Knockout DMEM (Life Technologies) with the addition of 2-mM L-Glutamine, 1× nonessential amino acids, 0.1-mM β-mercaptoethanol, 1000-U/mL ESGRO (Millipore), and 10% fetal bovine serum (Life Technologies) on a feeder layer of mouse embryonic fibroblasts. Cells were passaged with Trypsin (0.5% Trypsin, 0.1% chicken serum, 20-μg/mL EDTA, 10-μg/mL D-Glucose in PBS) and then replated at the required density.

In single suspension, 1 × 10^6^ cells were mixed with 2.5 μg of targeting vector and resuspended in 110 μl of solution R (Thermo Fisher Scientific). Cells were electroporated using the Neon transfection system (Thermo Fisher Scientific) (1400 V, 10 ms, 3 pulses) and expanded for clonal isolation in 75-μg/mL Hygromycin. Resistant colonies were manually picked, expanded, replica plated, and screened for homologous recombination events using primers, R26-5F1 and R26-5R1, for integration at the 5' end and primers, SV40-pA-F and ROSA3-R1, for integration at the 3' end. Heterozygous targeting and single copy integration was confirmed by PCR of the wild-type *Gt(ROSA26)Sor* locus using primers oIMR8545/oIMR8546 and Q-PCR using an in-house designed Taqman assay (Integrated DNA Technologies) and quantified relative to the reference gene, *Tfrc* (Thermo Fisher Scientific 4458366).

### Recombinase-mediated cassette exchange

In single suspension, 1 × 10^6^ mouse ES cells were electroporated with 5 μg of the exchange plasmid (Neon Transfection System, Thermo Fisher Scientific) (1400 V, 10 ms, 3 pulses) and selected in 210-μg/mL G418. Individual resistant colonies were isolated, expanded, and screened for the RMCE event using primers CAG-F and ExNeo2 to verify integration at the 5' end and primers CMV-IER and Rosa-3HR-R to verify integration at the 3' end. Individual exchanged clones were screened by Q-PCR for the presence of the Neomycin cassette using an in-house designed Taqman assay (Integrated DNA Technologies), and quantified relative to the reference gene, *Tfrc* (Thermo Fisher Scientific 4458366).

A total of 5 × 10^5^ human iPS cells were lipofected (Trans-IT, Mirus) with 1μg of the exchange plasmid and selected in 200-μg/mL G418. Individual resistant colonies were isolated, expanded and screened for the RMCE event using primers CAG-F or EF1a-F and ExNeo2 to verify integration at the 5' end and primers rbGpA-F1 and AAVS1-38-R1 to verify integration at the 3' end.

### Cre recombinase activation of expression

iPS cells were dissociated with Accutase (Merck, SCR005), plated at low density, and incubated with 1-μM TAT-Cre (Merck, SCR508) for 2 hours in the incubator. Individual colonies were isolated, expanded, and screened for the deletion event, first using primers which detect the intact allele (CAG-F or EF1a-F and ExNeo2) and then to screen clones negative for this PCR with deletion specific primers (CAG-F or EF1a-F and GFP-R2 [for GFP insertion] or mRFP1-R2b [for mRFP1 insertion]). Mosaic clones were plated at low density and individual clones isolated and genotyped again using the above strategy.

### Animals

Animal procedures were performed in accordance with UK Home Office Animal (Scientific Procedures) Act 1986 under project license PAA2AAE49, under review by the Clinical Medicine Animal Welfare and Ethical Review Body. Animals were housed in individually ventilated cages and provided with food and water ad libitum, maintained on a 12 hours/12 hours light cycle (150-200 lux).

Wild-type albino C57BL6/J females (Jax stock #000058) were superovulated and mated with albino C57BL/J males, and morulae were flushed from the uteri of plugged females on 2.5 days postcoitum. Embryos were cultured overnight to the blastocyst stage and microinjected with 8-12 ES cells, followed by surgical transfer into pseudopregnant CD1 recipients. The resulting chimeric males were mated with C57BL/6J females and offspring assessed for fluorescent protein expression or for LacZ expression by whole-mouse X-Gal staining.

### Validation of CRISPRa and CRISPRi cell lines

For the CRISPRa sgRNAs, complimentary oligonucleotides containing the sgRNA target sequences were annealed and cloned into the BbsI site of sgRNA(MS2) cloning backbone (Addgene #42230) as previously described. DNA templates for in vitro transcription were generated from these plasmids using primers T7-NGN2 or T7-HHEX (forward primers with a 5' extension corresponding to a T7 polymerase binding site) with a reverse primer, gRNA-R binding downstream of the mature sgRNA sequence. sgRNAs were prepared by in vitro transcription using the MEGAshortscript T7 Transcription Kit (Thermo Fisher Scientific, #AM1354) and purified using the MEGAclear Transcription Clean-up Kit (Thermo Fisher Scientific, #AM1908). For the CRISPRi sgRNAs, synthetic sgRNA were designed using the CRISPOR tool (https://crispor.gi.ucsc.edu/) and were chemically synthesized by Synthego.

A total of 1 × 10^5^ KOLF-AAVS1-SAM or KOLF-AAVS1-KRAB iPS cells were seeded in 12-well plates and transfected with 1-μg sgRNA using RNAiMAX (Thermo Fisher Scientific) according to the manufacturer’s instructions. Where 2 sgRNAs were used, 500 ng of each sgRNA was lipofected. Cells were cultured for either 48 or 96 hours prior to RNA extraction using TRIzol reagent (Themofisher Scientific, #15596026), and 400 ng of RNA was reverse transcribed using the qPCRBIO cDNA synthesis kit (PCR Biosystems, #PB30.11-02) according to manufacturer’s instructions. Quantitative PCR was carried out using PowerUp SYBR Green Master Mix (Applied Biosystems, #A25742) according to manufacturer instructions. qPCR parameters were quantified using CFX96 Touch Real-Time PCR Detection System (BioRad). Expression fold-change relative to parallel transfections with a nontargeting sgRNA was calculated using *TBP* as a reference transcript.

For immunostaining of NGN2, cells were washed once with PBS and fixed in 4% paraformaldehyde in PBS for 15 minutes at room temperature. The cells were then washed 3 times in PBS and permeabilized with 0.2% Triton X-100/PBS for 10 minutes at room temperature. The cell were incubated in blocking buffer (5% fish gelatin, 0.3-M glycine in PBS) for 1 hour at room temperature. The cells were incubated with Anti-Neurogenin2 antibody (1:200 dilution, Cell Signaling Technology, #rabbit mAB#13144) and mouse anti-human TRA-1-81 dyLight488 monoclonal antibody (1:500 dilution, Invitrogen, #MA1-024-D488) in PBS at 4 °C overnight. The following day, the cells were washed with PBS and stained with Goat-anti rabbit Alexa Fluor568 (1:500 dilution, Invitrogen, #A-11036) at room temperature and in the dark for 1 hour. After 3 more washes in PBS, the coverslips were mounted on a clean glass slide with DAPI-containing fluoroshield (Sigma, #F6057-20ML) and visualized on an SP8 LIGHTINING confocal microscope (Leica).

For FACS analysis of the pluripotency markers, cells were washed once with PBS and dissociated into a single-cell suspension with Accutase (Merck, SCR005). The cells were then washed with PBS and processed according to the manufacturer’s instructions using the BD Human Pluripotent Stem Cell Transcription Factor Analysis Kit (BD Biosciences). Minor modifications were performed by adding FITC Mouse anti-SSEA-4 (Clone MC813-70, BD Biosciences, #560126) to the specific antibody panel and FITC Mouse IgG3, κ Isotype Control (Clone J606, BD Biosciences, Cat. No. 555578) to the panel of antibody controls. An additional anti-mouse beads single stain control for FITC Mouse anti-SSEA-4 was generated to adjust scatter and fluorescence settings and calculate compensation. FlowJo software (BD Biosciences, version 10.6.2) was used for analysis. Median Fluorescence Intensity following the transfection of targeting sgRNA relative to nontargeting sgRNA was used to demonstrate the reduction in protein levels.

Heterozygous targeting and single copy integration was confirmed by PCR of the wild-type *AAVS1* locus using primers AAVS1-37-F1/AAVS1-39-R1 and Q-PCR using an in-house designed Taqman assay (Integrated DNA Technologies) against the CAG promoter and quantified relative to the reference gene, *RPPH1* (Thermofisher Scientific 4403328). For karyotype analysis, copy number estimation was performed using the QDNASeq package in R (version 3.5.1) to analyze low-pass sequencing data from genomic DNA prepared from KOLF-AAVS-SAM and KOLF-AAVS-KRAB cells according to the method described.^62^

## Supplementary material

Supplementary material is available at *Stem Cells* online.

sxae092_suppl_Supplementary_Figures_1-11

## Data Availability

The data underlying this article are available in the article and in its online supplementary material.
