# Supplementary material for: Optimizing approaches for targeted integration of transgenic cassettes by integrase-mediated cassette exchange in mouse and human stem cells
Source: Stem Cells. 2025 Jan 8;43(1):sxae092. doi: 10.1093/stmcls/sxae092 (PMC11740728; doi:10.1093/stmcls/sxae092)
Supplement: sxae092_suppl_Supplementary_Figures_1-11 [file sxae092_suppl_supplementary_figures_1-11.pdf]

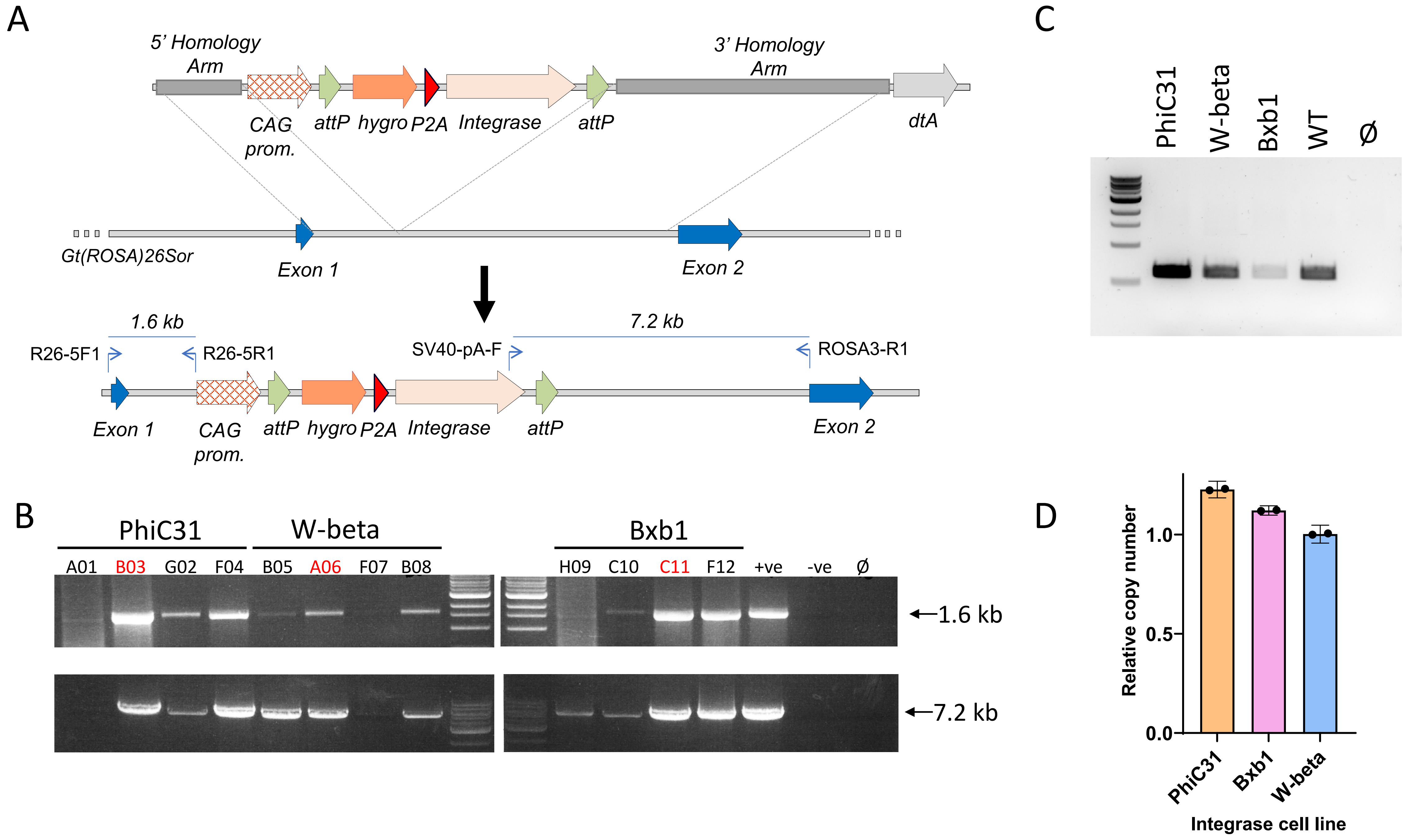
A
5' Homology Arm
3' Homology Arm
CAG prom.
attP
hygro
P2A
Integrase
attP
dtA
Gt(ROSA)26Sor
Exon 1
Exon 2
1.6 kb
7.2 kb
R26-5F1
R26-5R1
SV40-pA-F
ROSA3-R1
Exon 1
CAG prom.
attP
hygro
P2A
Integrase
attP
Exon 2
B
PhiC31
W-beta
Bxb1
A01 B03 G02 F04 B05 A06 F07 B08
H09 C10 C11 F12 +ve -ve Ø
1.6 kb
7.2 kb
C
PhiC31
W-beta
Bxb1
WT
Ø
D
Relative copy number
0.0
0.5
1.0
PhiC31
Bxb1
W-beta
Integrase cell line

Fig. S2

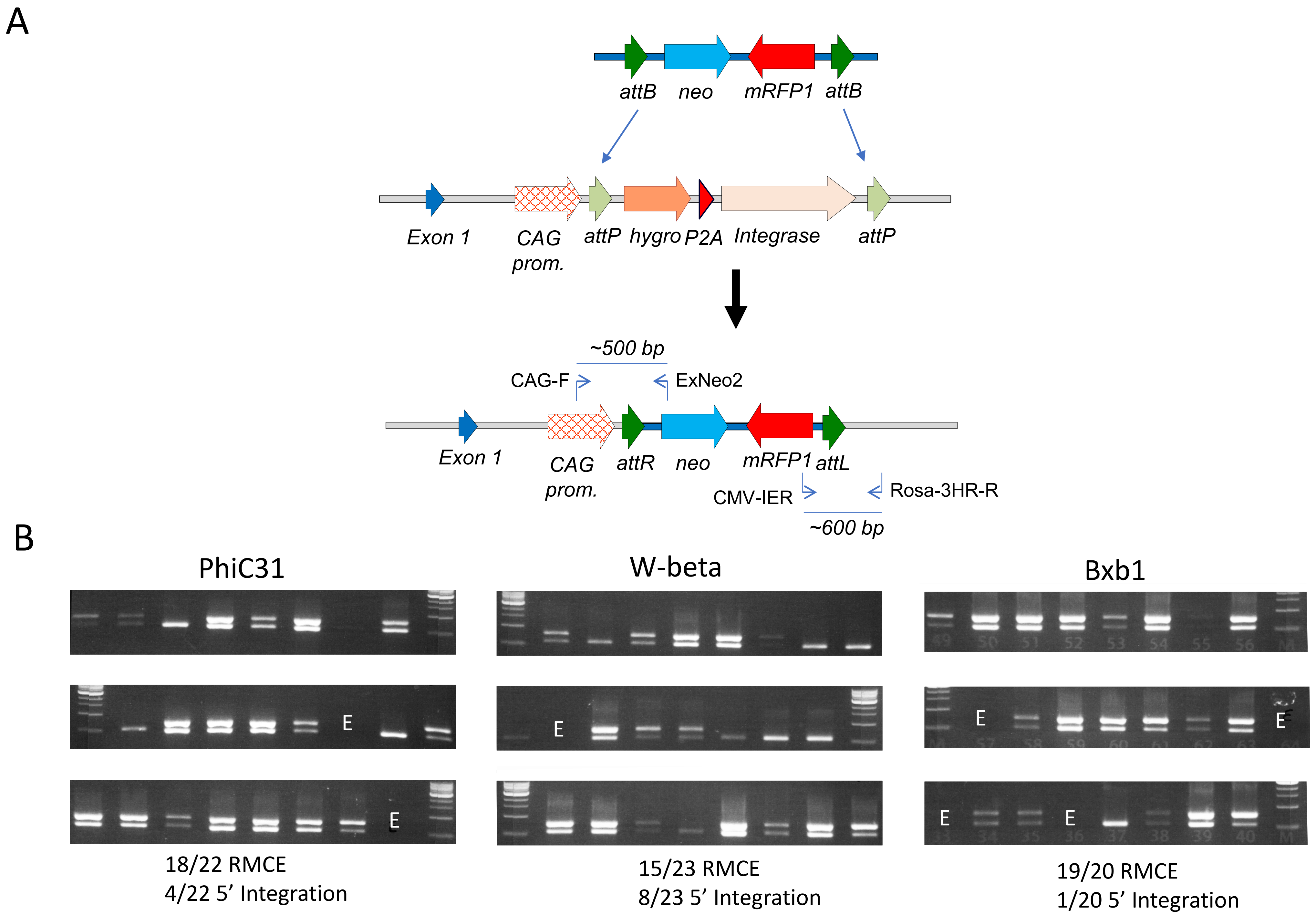
A
attB
neo
mRFP1
attB
Exon 1
CAG
prom.
attP
hygro
P2A
Integrase
attP
~500 bp
CAG-F
ExNeo2
Exon 1
CAG
prom.
attR
neo
mRFP1
attL
CMV-IER
Rosa-3HR-R
~600 bp
B
PhiC31
W-beta
Bxb1
E
E
E
E
E
E
E
18/22 RMCE
4/22 5’ Integration
15/23 RMCE
8/23 5’ Integration
19/20 RMCE
1/20 5’ Integration

Fig. S3

System	Clone	attR	Clone	attL
**W-Beta**	1	TGTGATATTTGTAACGGTACTTCCAACAGCTGGCGTTTCAGTGGATCAAAC<br>GCGCTGTTCTCCTCTTCCTCATCTCCGGGCCTTTCGACGTGCAGCCAATATG<br>GGATCGGCCATT	1	ATCCAAGGTAGCGTCAACGATAGGTGTAACTGTCGTGTTTATCAC<br>GGTACCCAATAACCAATGAATATTTGATTCGAACTCGAGATGCAT<br>CCGCG
**W-Beta**	2	TGTGATATTTGTAACGGTACTTCCAACAGCTGGCGTTTCAGTGGATCAAAC<br>GCGCTGTTCTCCTCTTCCTCATCTCCGGGCCTTTCGACGTGCAGCCAATATG<br>GGATCGGCCATT	2	CGATCCAAGGTAGCGTCAACGATAGGTGTAACTGTCGTGTTTATC<br>ACGGTACCCAATAACCAATGAATATTTGATTCGAACTCGAGATGC<br>ATCCG
**W-Beta**	3	TGTGATATTTGTAACGGTACTTCCAACAGCTGGCGTTTCAGTGGATCAAAC<br>GCGCTGTTCTCCTCTTCCTCATCTCCGGGCCTTTCGACGTGCAGCCAATATG<br>GGATCGGCCATT	3	CGATCCAAGGTAGCGTCAACGATAGGTGTAACTGTCGTGTTTATC<br>ACGGTACCCAATAACCAATGAATATTTGATTCGAACTCGAGATGC<br>ATCCG
**W-Beta**	4	TGTGATATTTGTAACGGTACTTCCAACAGCTGGCGTTTCAGTGGATCAAAC<br>GCGCTGTTCTCCTCTTCCTCATCTCCGGGCCTTTCGACGTGCAGCCAATATG<br>GGATCGGCCATT	4	CGATCCAAGGTAGCGTCAACGATAGGTGTAACTGTCGTGTTTATC<br>ACGGTACCCAATAACCAATGAATATTTGATTCGAACTCGAGATGC<br>ATCCG
**PhiC31**	1	GTTTCGAAGTAGTGCCCCAACTGGGGTAACCTTTGGGCTCCCCGGGCGCGT<br>ACTCCACGGATCAAACGCGCTGTTCTCCTCTTCCTCATCTCCGGGCCTTTCG<br>ACCTGCAGCCAATATGGGATCGGCCATTGAACAAGAT	1	CGATCCCCGCGGTGCGGGTGCCAGGGCGTGCCCTTGAGTTCTCTC<br>AGTTGGGGGCGTAGCTCGAGGGGCGCGCCGGGATCCCTAGACCG<br>CGGGGC
**PhiC31**	2	GTTTCGAAGTAGTGCCCCAACTGGGGTAACCTTTGGGCTCCCCGGGCGCGT<br>ACTCCACGGATCAAACGCGCTGTTCTCCTCTTCCTCATCTCCGGGCCTTTCG<br>ACCTGCAGCCAATATGGGATCGGCCATTGAACAAGAT	2	CGATCCCCGCGGTGCGGGTGCCAGGGCGTGCCCTTGAGTTCTCTC<br>AGTTGGGGGCGTAGCTCGAGGGGCGCGCCGGGATCCCTAGACCG<br>CGGG
**PhiC31**	3	GTTTCGAAGTAGTGCCCCAACTGGGGTAACCTTTGGGCTCCCCGGGCGCGT<br>ACTCCACGGATCAAACGCGCTGTTCTCCTCTTCCTCATCTCCGGGCCTTTCG<br>ACCTGCAGCCAATATGGGATCGGCCATTGAACAAGAT	3	CGATCCCCGCGGTGCGGGTGCCAGGGCGTGCCCTTGAGTTCTCTC<br>AGTTGGGGGCGTAGCTCGAGGGGCGCGCCGGGATCCCTAGACCG<br>CGGGGC
**PhiC31**	4	GTTTCGAAGTAGTGCCCCAACTGGGGTAACCTTTGGGCTCCCCGGGCGCGT<br>ACTCCACGGATCAAACGCGCTGTTCTCCTCTTCCTCATCTCCGGGCCTTTCG<br>ACCTGCAGCCAATATGGGATCGGCCATTGAACAAGAT	4	CGATCCCCGCGGTGCGGGTGCCAGGGCGTGCCCTTGAGTTCTCTC<br>AGTTGGGGGCGTAGCTCGAGGGGCGCGCCGGGATCCCTAGACCG<br>CGGGGC
**Bxb1**	1	GTTTCGAAGTCGTGGTTTGTCTGGTCAACCACCGCGGTCTCCGTCGTCAGG<br>ATCATCCGGGCGGATCAAACGCGCTGTTCTCCTCTTCCTCATCTCCGGGCCT<br>TTCGACGTGCAGCCAATATGGGATCGGCCATTGAACA	1	GCGATCCTCGGCCGGCTTGTCGACGACGGCGGTCTCAGTGGTGTA<br>CGGTACAAACCCCGACTTCGAACTCGAGGGGCGCGCCGGGATCC<br>CTAGAC
**Bxb1**	2	GTTTCGAAGTCGTGGTTTGTCTGGTCAACCACCGCGGTCTCCGTCGTCAGG<br>ATCATCCGGGCGGATCAAACGCGCTGTTCTCCTCTTCCTCATCTCCGGGCCT<br>TTCGACGTGCAGCCAATATGGGATCGGCCATTGAACA	2	GCGATCCTCGGCCGGCTTGTCGACGACGGCGGTCTCAGTGGTGTA<br>CGGTACAAACCCCGACTTCGAACTCGAGGGGCGCGCCGGGATCC<br>CTAGA
**Bxb1**	3	GTTTCGAAGTCGTGGTTTGTCTGGTCAACCACCGCGGTCTCCGTCGTCAGG<br>ATCATCCGGGCGGATCAAACGCGCTGTTCTCCTCTTCCTCATCTCCGGGCCT<br>TTCGACGTGCAGCCAATATGGGATCGGCCATTGAACA	3	GCGATCCTCGGCCGGCTTGTCGACGACGGCGGTCTCAGTGGTGTA<br>CGGTACAAACCCCGACTTCGAACTCGAGGGGCGCGCCGGGATCC<br>CTAGAC
**Bxb1**	4	GTTTCGAAGTCGTGGTTTGTCTGGTCAACCACCGCGGTCTCCGTCGTCAGG<br>ATCATCCGGGCGGATCAAACGCGCTGTTCTCCTCTTCCTCATCTCCGGGCCT<br>TTCGACGTGCAGCCAATATGGGATCGGCCATTGAACA	4	GCGATCCTCGGCCGGCTTGTCGACGACGGCGGTCTCAGTGGTGTA<br>CGGTACAAACCCCGACTTCGAACTCGAGGGGCGCGCCGGGATCC<br>CTAGA

Fig. S4

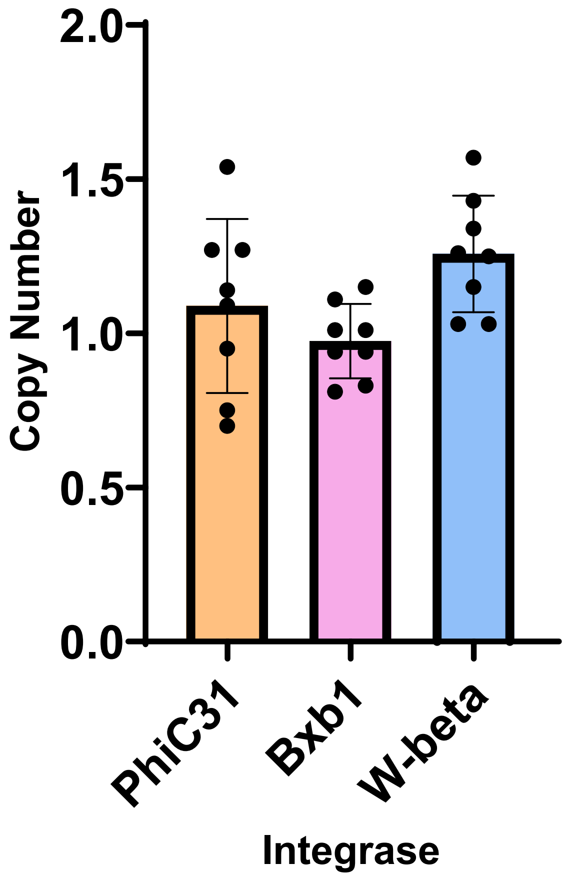

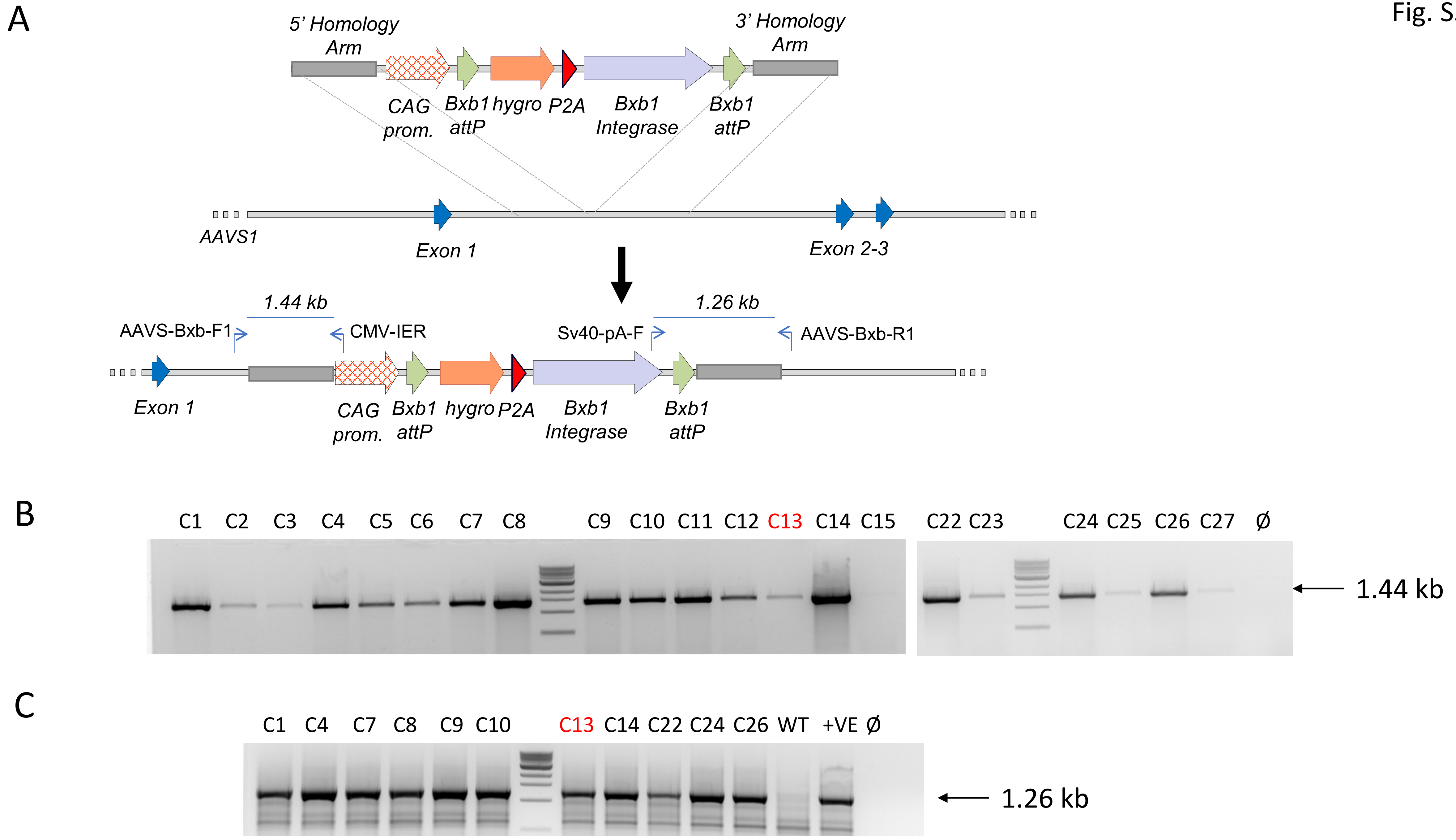
A
5' Homology Arm
3' Homology Arm
CAG prom.
Bxb1 attP
hygro
P2A
Bxb1 Integrase
Bxb1 attP
AAVS1
Exon 1
Exon 2-3
1.44 kb
1.26 kb
AAVS-Bxb-F1
CMV-IER
Sv40-pA-F
AAVS-Bxb-R1
Exon 1
CAG prom.
Bxb1 attP
hygro
P2A
Bxb1 Integrase
Bxb1 attP
B
C1 C2 C3 C4 C5 C6 C7 C8 C9 C10 C11 C12 C13 C14 C15 C22 C23 C24 C25 C26 C27 Ø
1.44 kb
C
C1 C4 C7 C8 C9 C10 C13 C14 C22 C24 C26 WT +VE Ø
1.26 kb

Fig. S6

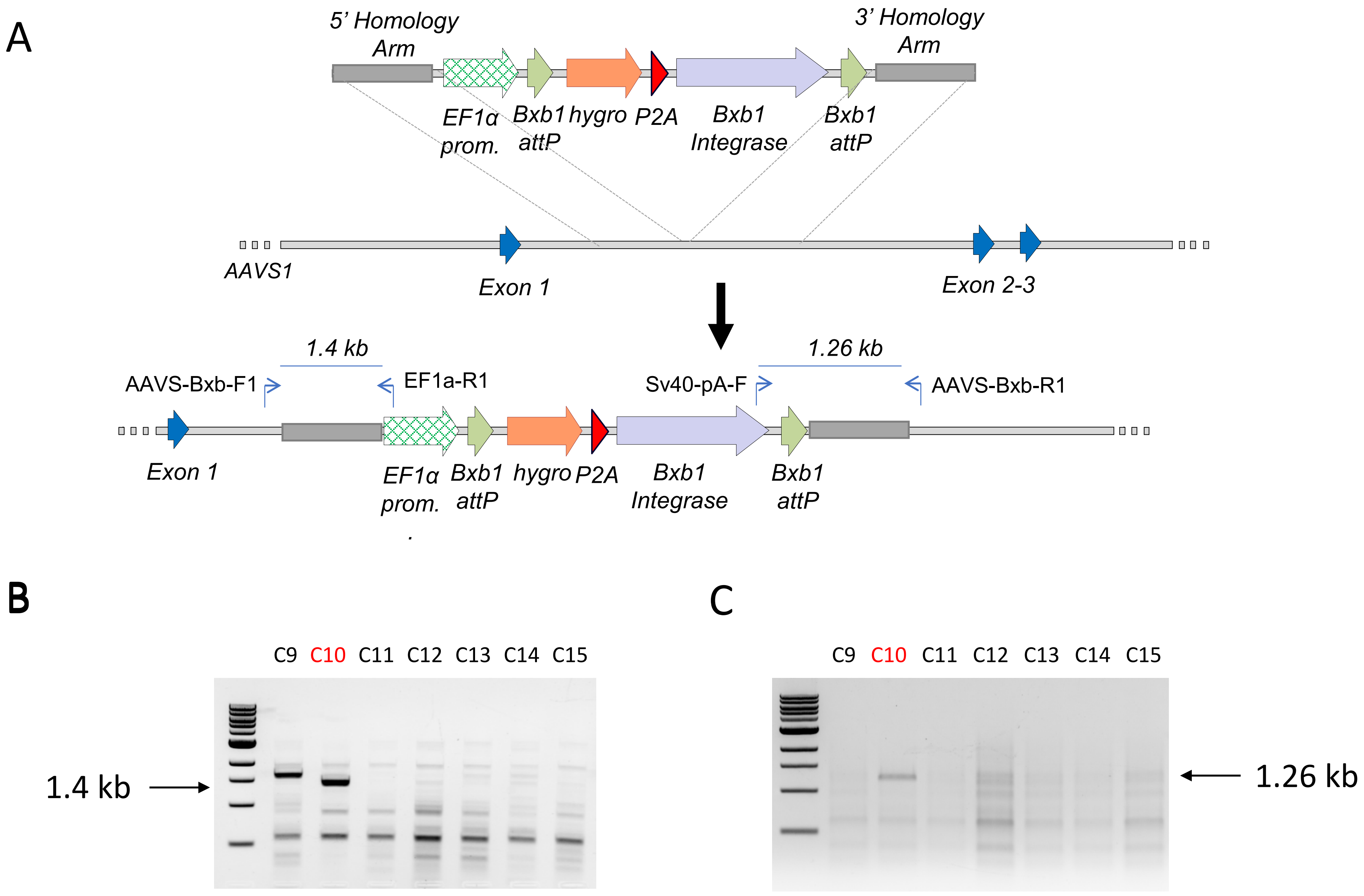
A
5' Homology Arm
3' Homology Arm
EF1α prom.
Bxb1 attP
hygro
P2A
Bxb1 Integrase
Bxb1 attP
AAVS1
Exon 1
Exon 2-3
1.4 kb
1.26 kb
AAVS-Bxb-F1
EF1a-R1
Sv40-pA-F
AAVS-Bxb-R1
Exon 1
EF1α prom.
Bxb1 attP
hygro
P2A
Bxb1 Integrase
Bxb1 attP
B
C9
C10
C11
C12
C13
C14
C15
1.4 kb
C
C9
C10
C11
C12
C13
C14
C15
1.26 kb

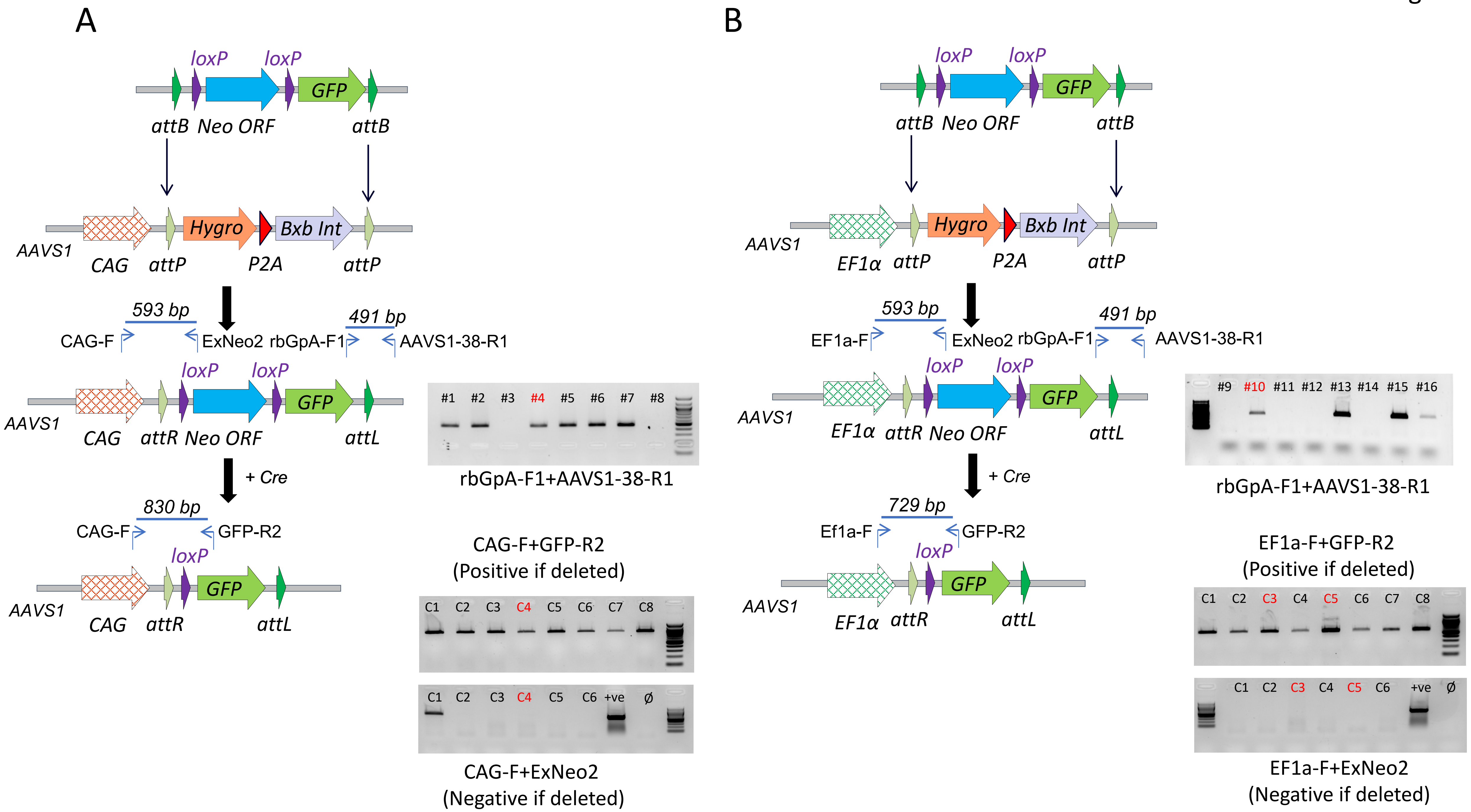
A
loxP
loxP
GFP
attB
Neo ORF
attB
AAVS1
CAG
attP
Hygro
P2A
Bxb Int
attP
593 bp
491 bp
CAG-F
ExNeo2
rbGpA-F1
AAVS1-38-R1
loxP
loxP
GFP
AAVS1
CAG
attR
Neo ORF
attL
+ Cre
830 bp
CAG-F
GFP-R2
loxP
GFP
AAVS1
CAG
attR
attL
#1 #2 #3 #4 #5 #6 #7 #8
rbGpA-F1+AAVS1-38-R1
CAG-F+GFP-R2
(Positive if deleted)
C1 C2 C3 C4 C5 C6 C7 C8
C1 C2 C3 C4 C5 C6 +ve ø
CAG-F+ExNeo2
(Negative if deleted)
B
loxP
loxP
GFP
attB
Neo ORF
attB
AAVS1
EF1α
attP
Hygro
P2A
Bxb Int
attP
593 bp
491 bp
EF1a-F
ExNeo2
rbGpA-F1
AAVS1-38-R1
loxP
loxP
GFP
AAVS1
EF1α
attR
Neo ORF
attL
+ Cre
729 bp
Ef1a-F
GFP-R2
loxP
GFP
AAVS1
EF1α
attR
attL
#9 #10 #11 #12 #13 #14 #15 #16
rbGpA-F1+AAVS1-38-R1
EF1a-F+GFP-R2
(Positive if deleted)
C1 C2 C3 C4 C5 C6 C7 C8
C1 C2 C3 C4 C5 C6 +ve ø
EF1a-F+ExNeo2
(Negative if deleted)

Fig. S8

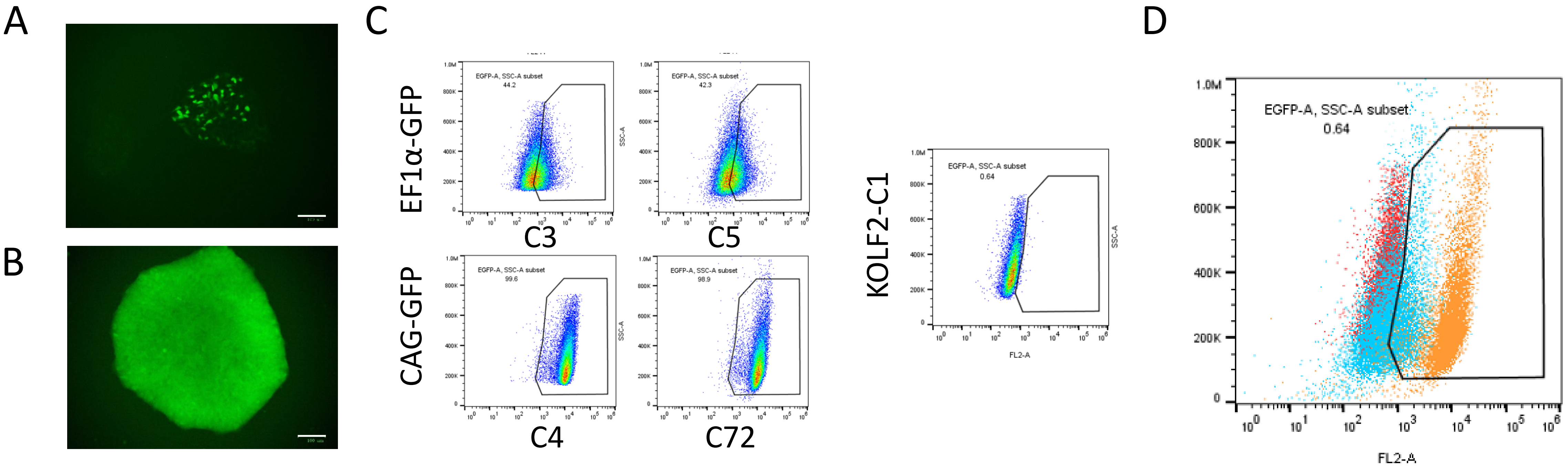

A
B
C
EF1α-GFP
EGFP-A, SSC-A subset
44.2
C3
EGFP-A, SSC-A subset
42.3
C5
CAG-GFP
EGFP-A, SSC-A subset
99.6
C4
EGFP-A, SSC-A subset
98.9
C72
KOLF2-C1
EGFP-A, SSC-A subset
0.64
FL2-A
SSC-A
D
EGFP-A, SSC-A subset
0.64
1.0M
800K
600K
400K
200K
0
FL2-A

Fig. S9

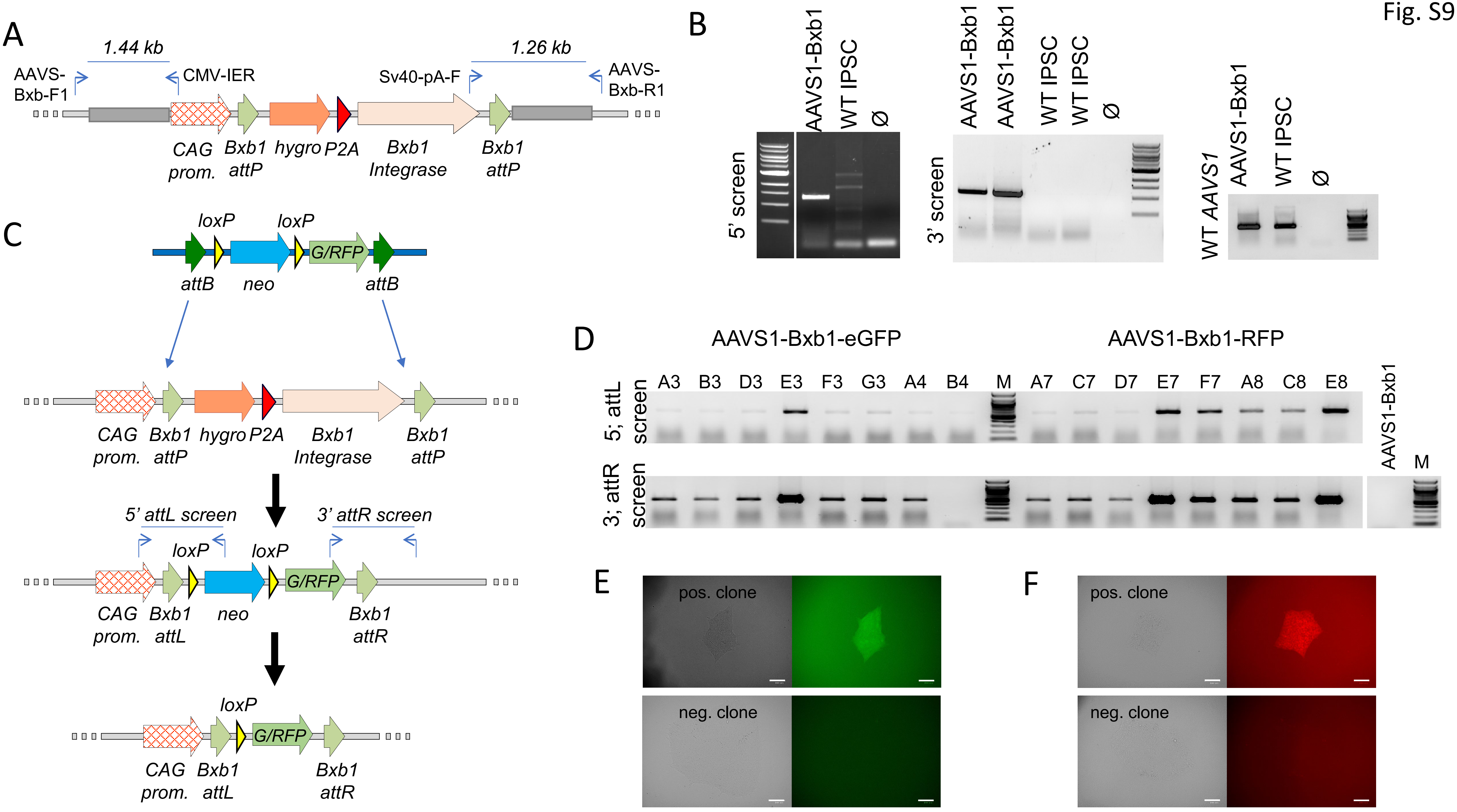
A
1.44 kb
1.26 kb
AAVS-Bxb-F1
CMV-IER
Sv40-pA-F
AAVS-Bxb-R1
CAG prom.
Bxb1 attP
hygro
P2A
Bxb1 Integrase
Bxb1 attP
B
5' screen
AAVS1-Bxb1
WT IPSC
Ø
3' screen
AAVS1-Bxb1
AAVS1-Bxb1
WT IPSC
WT IPSC
Ø
WT AAVS1
AAVS1-Bxb1
WT IPSC
Ø
C
loxP
loxP
attB
neo
G/RFP
attB
CAG prom.
Bxb1 attP
hygro
P2A
Bxb1 Integrase
Bxb1 attP
5' attL screen
3' attR screen
loxP
loxP
CAG prom.
Bxb1 attL
neo
G/RFP
Bxb1 attR
loxP
CAG prom.
Bxb1 attL
G/RFP
Bxb1 attR
D
AAVS1-Bxb1-eGFP
AAVS1-Bxb1-RFP
5; attL screen
3; attR screen
A3
B3
D3
E3
F3
G3
A4
B4
M
A7
C7
D7
E7
F7
A8
C8
E8
AAVS1-Bxb1
M
E
pos. clone
neg. clone
F
pos. clone
neg. clone

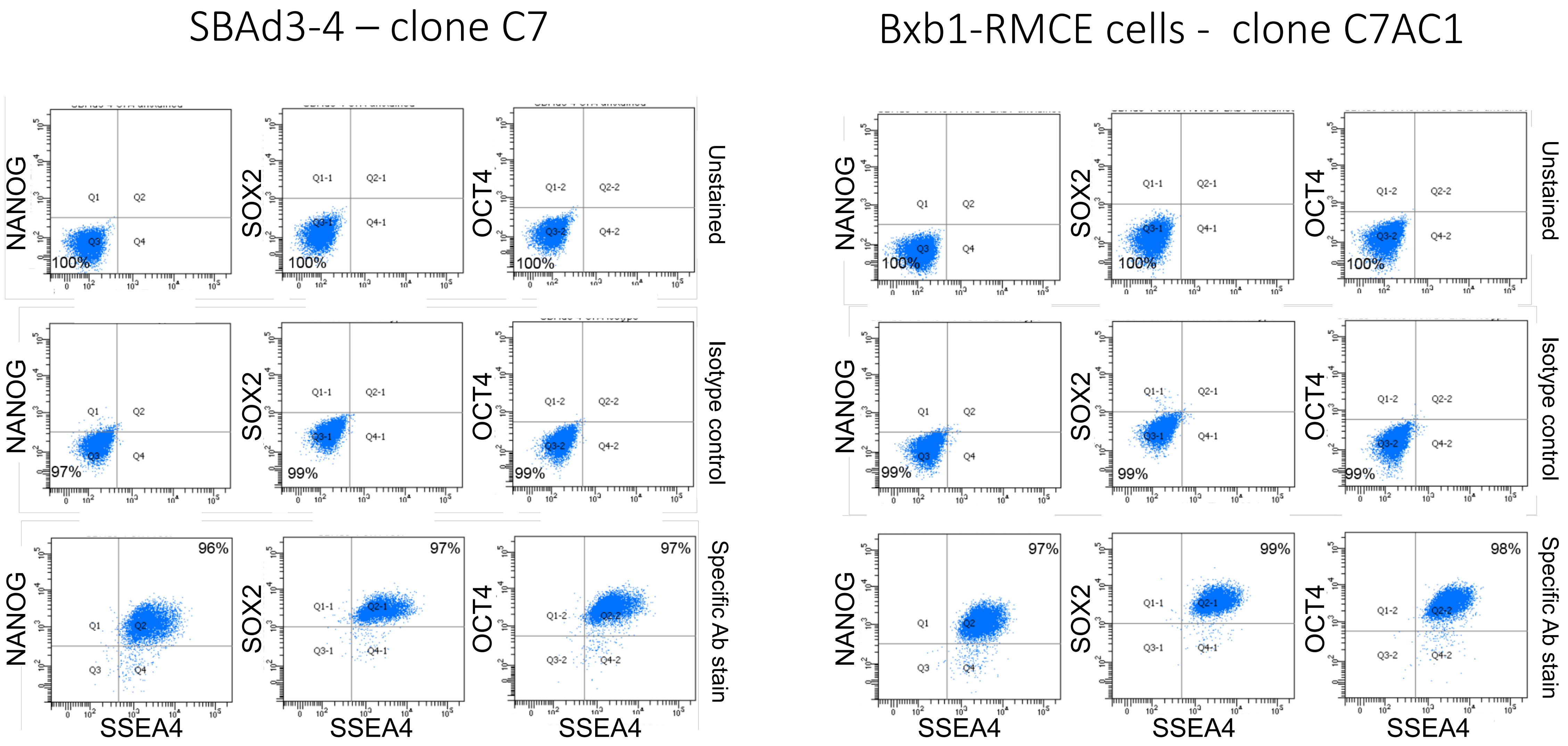
SBAd3-4 – clone C7
Bxb1-RMCE cells - clone C7AC1
Unstained
Isotype control
Specific Ab stain
NANOG
SOX2
OCT4
SSEA4
100%
97%
99%
96%
98%

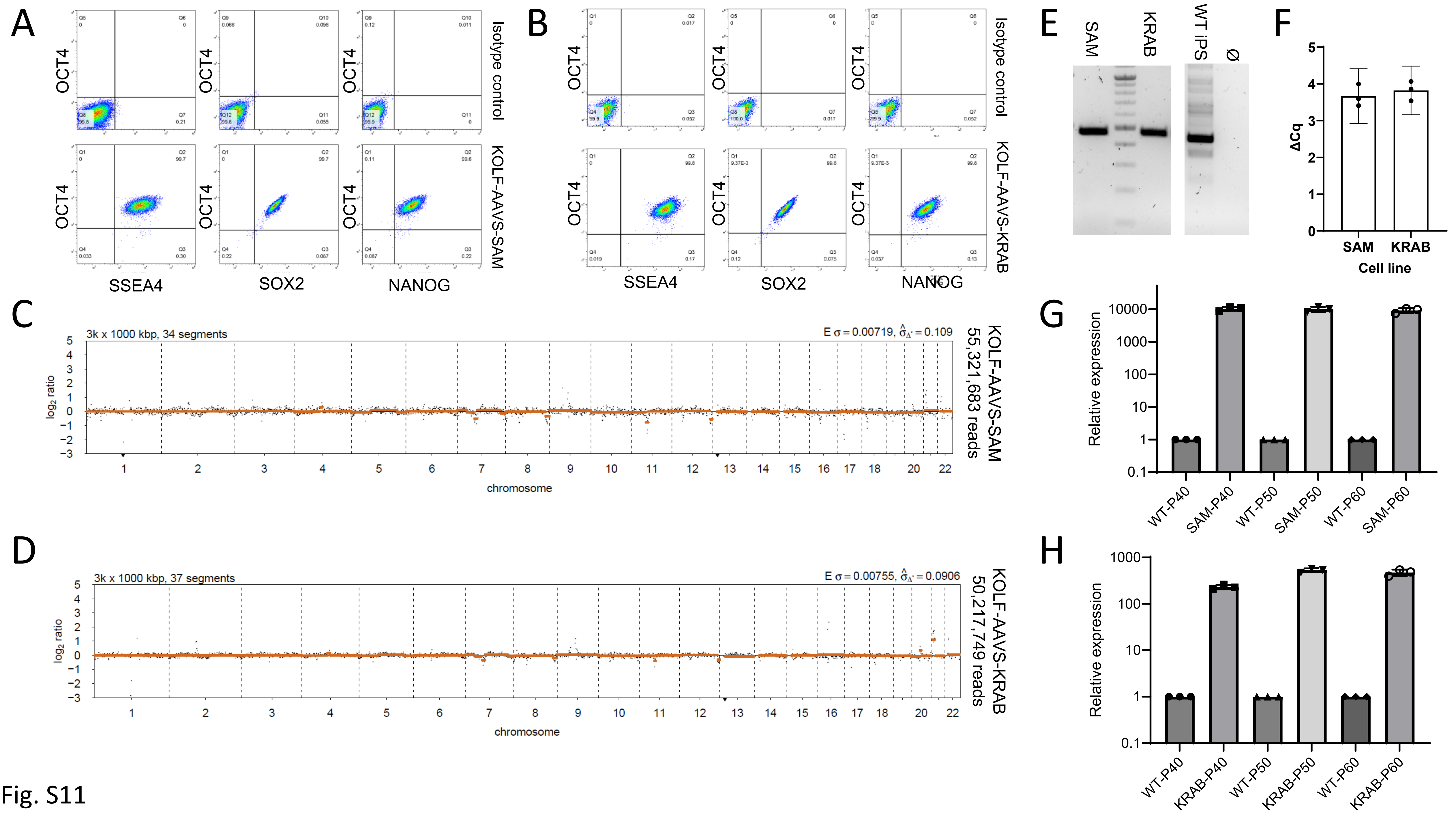
A
B
C
D
E
F
G
H
Isotype control
KOLF-AAVS-SAM
KOLF-AAVS-KRAB
OCT4
SSEA4
SOX2
NANOG
3k x 1000 kbp, 34 segments
3k x 1000 kbp, 37 segments
log2 ratio
chromosome
55,321,683 reads
50,217,749 reads
SAM
KRAB
WT iPS
Ø
ΔCq
Cell line
Relative expression
WT-P40
SAM-P40
WT-P50
SAM-P50
WT-P60
SAM-P60
KRAB-P40
KRAB-P50
KRAB-P60

Fig. S11
